# Sampling Frequency Requirements for Estimating Physical Activity Metrics from Wearable Accelerometer Data

**DOI:** 10.3390/s26206652

**Published:** 2026-10-21

**Authors:** Marcin Straczkiewicz

**Affiliations:** Department of Measurement and Electronics, AGH University of Krakow, 30-059 Krakow, Poland; mstr@agh.edu.pl

**Keywords:** wearable accelerometry, sampling frequency, physical activity, wearable sensors, signal processing, digital biomarkers, population health, sensor placement

## Abstract

**Highlights:**

**What are the main findings?**

- The sampling frequency required to quantify physical activity depends on the activity, sensor location, and the accelerometry-derived metric.
- Lower sampling frequencies can preserve estimates for many movement conditions, whereas more dynamic activities and selected metrics are more sensitive to frequency reduction.

**What are the implications of the main findings?**

- Higher accelerometer sampling frequencies are not universally necessary for reliable physical activity quantification.
- Sampling frequency can be selected according to the intended outcome, providing a practical trade-off between signal fidelity, data volume, and device resource requirements.

**Abstract:**

Accelerometers are widely used to quantify physical activity in population health research, yet the sampling frequency required to preserve commonly reported measures remains uncertain. We evaluated sampling frequency using 315.4 h of labelled tri-axial accelerometer data from 321 participants across 11 publicly available datasets. Signals were harmonized to a 50 Hz reference and downsampled to 40, 30, 20, 10, and 5 Hz using anti-aliased resampling. We compared Monitor-Independent Movement Summary (MIMS), Euclidean Norm Minus One (ENMO), Activity Index (AI), dominant spectral frequency, and retained spectral power across activities and sensor locations. For MIMS, 20 Hz remained within the prespecified 5% error margin for many walking, stair-climbing, cycling, jumping, household activities, and motorized-transportation conditions, whereas running exceeded this margin at 20 Hz at lower-limb and trunk locations. At 10 Hz, errors increased for most ambulatory activities, and only about 60–72% of reference spectral power was retained for walking and running, despite preservation of their dominant movement frequencies. MIMS error was not associated with age, sex/gender, or BMI, although small sex/gender associations were observed for ENMO and AI. Sampling-frequency requirements, therefore, depend on the metric, activity, and sensor location, supporting fit-for-purpose selection rather than use of a single universal sampling rate.

## 1. Introduction

Raw accelerometer data are increasingly used to derive metrics of movement intensity in clinical and population health studies. These measures do not directly quantify physical activity behavior or energy expenditure, but rather summarize selected properties of the recorded acceleration signal that can subsequently be used to characterize movement. Sampling frequency is one of the fundamental data acquisition parameters that may influence these estimates. Previous studies have shown that sampling frequency can alter raw acceleration summaries and activity classification performance, but have generally focused on particular devices, populations, wear locations, or algorithms [1,2,3,4,5]. It therefore remains unclear whether a sampling frequency sufficient to recognize an activity may be adequate to preserve the acceleration magnitude or spectral information used to derive quantitative movement measures.

To address this gap, we applied a harmonized downsampling framework to labelled raw accelerometer data from 11 publicly available datasets. Signals collected using smartphones, smartwatches, and wearable accelerometers were standardized to a common 50 Hz reference and progressively downsampled using an identical anti-aliasing procedure, allowing paired comparisons within the same activity bout. We evaluated preservation of dominant movement frequency and spectral power, together with three established physical activity metrics, Monitor-Independent Movement Summary (MIMS), Euclidean Norm Minus One (ENMO), and Activity Index (AI). These metrics were selected because they rely on different signal properties and processing procedures: MIMS incorporates signal reconstruction, filtering, rectification, and integration [6]; ENMO summarizes gravity-adjusted vector magnitude [7]; and AI quantifies short-term acceleration variability [8]. We therefore examined whether a sampling frequency sufficient to preserve one metric would also be sufficient for the others across different activities and sensor locations. We focused on measures that could be calculated using a common processing framework across heterogeneous sensing devices, rather than manufacturer-specific activity counts whose values depend on device-specific processing and acquisition parameters [1].

In this work, our objective was to determine how sampling frequency affects these estimates and to identify the lowest frequency that preserves them within a predefined error margin. We hypothesized that the sampling frequency required to preserve accelerometry-derived estimates would vary according to the metric, activity, and sensor location. In particular, we expected faster and more dynamic activities to require higher sampling frequencies than stationary or slowly varying behaviors. We further expected that the predominant movement frequency could remain identifiable at sampling frequencies that no longer preserve broader spectral content or movement-intensity estimates. As a secondary analysis, we also examined whether downsampling-related estimation error was systematically associated with participant age, sex/gender, or BMI after accounting for activity, sensor location, sampling frequency, and source dataset.

## 2. Materials and Methods

### 2.1. Population and Data

We aggregated data from 11 publicly available datasets containing raw tri-axial accelerometer measurements and corresponding activity labels. Activity labels were used to identify known activity bouts and to stratify comparisons by activity type. The consolidated dataset consisted of measurements collected from 321 healthy individuals (Table 1).

The availability of detailed demographic and anthropometric information varied across the studies. Specific characteristics, including sex/gender, height, weight, and Body Mass Index (BMI), were consistently reported in *DaLiAc*, *IWSDC*, *MobiAct*, *MotionSense*, *RealWorld*, *SisFall*, and *SPADES*, whereas these details were incomplete or not provided in the *CHA*, *PARDUSS*, *HAPT*, and *SpeedBreaker* datasets. Cumulatively, sex/gender was available for 248 subjects (154 male); age was available for 230 subjects (range: 16.0–75.0 years; mean ± SD: 30.1 ± 12.3 years). Height was available for 229 subjects (range: 147.3–196.0 cm; 172.9 ± 9.8 cm), and weight was available for 229 subjects (range: 41.0–140.6 kg; 73.0 ± 16.6 kg). BMI was calculated for 229 subjects (range: 17.0–39.8 kg·m^−2^; 24.2 ± 4.2 kg·m^−2^). Overall, complete information on demographic and anthropometric characteristics was available for 226 subjects (70.4%).

The protocols across datasets captured a diverse set of physical activities. The majority of the studies focused on fundamental ambulatory and stationary behaviors, including flat walking, ascending and descending stairs, sitting, and standing. More vigorous physical activities, such as jogging, running, cycling, and jumping, were systematically recorded in *DaLiAc*, *PARDUSS*, *RealWorld*, and *SPADES*. Specialized functional tasks, including typing, handwriting, eating, drinking, and household chores like sweeping or washing dishes were captured in the *CHA*, *DaLiAc*, and *SPADES* datasets.

The *IWSDC* dataset included locomotion and motorized transportation, whereas *SpeedBreaker* was retained specifically as a contrasting measurement method. Unlike the predominantly structured human activities represented in the other datasets, it contained unconstrained smartphone measurements during motorized transportation. Although motorized transportation does not represent a physical activity *per se*, these recordings provide a low-dominant-frequency but vibration-rich condition for examining sampling-frequency effects outside structured human movement.

Measurement conditions and experimental protocols varied across datasets, ranging from prescribed activities performed under controlled laboratory conditions to structured field protocols and more naturalistic measurements. Detailed descriptions of the original data collection settings, activity execution, trial structure, and activity annotation are provided in Supplementary Table S2.

Data acquisition was conducted using commercial consumer-grade smartphones, smartwatches, and research-grade wearable accelerometers, including SHIMMER (Dublin, Ireland), ActiGraph GT3X+ and GT9X (Ametris, Pensacola, FL, USA). One dataset, *SisFall*, utilized a custom-developed device. The investigated datasets consisted of data collected on the thigh, wrist, waist, chest, and arm. In the free-living *SpeedBreaker* dataset, sensor locations were left intentionally unspecified to replicate unconstrained, real-world device usage.

Finally, technical data acquisition parameters varied among the hardware, with approximate sampling rates ranging from a baseline of 50 Hz up to 204.8 Hz. Among the studies detailing specific hardware configurations, amplitude resolutions spanned from 8 to 13 bits, and the measurement range was between 6 and ±16 g.

### 2.2. Data Preprocessing

Raw three-dimensional accelerometer data served as the primary input for all analyses. Measurements provided in standard SI units were converted to gravitational units (g) utilizing the standard definition of $1\;g=9.80665{\;m/s}^{2}$. To facilitate interpretation, investigated physical activities were consolidated into the following standardized categories: *flat walking*, including any walking other than stair climbing; *stair climbing*, including walking upstairs and downstairs; *running*, including running and jogging; *cycling*, including slow and fast cycling; *jumping*, including jumping forward, backward, and rope jumping; *motorized transportation*, including driving a car, bus, rickshaw, etc.; *household activities*, including sweeping, washing dishes, vacuuming, folding towels, filling shelves, etc.; and *stationary activities*, including standing still, sitting still, working on a computer, watching TV, etc. Sensor placements were similarly grouped into four standardized body regions: *lower limb*, including thigh; *trunk*, including waist and chest; *upper limb*, including arm and wrist; and *unconstrained*.

**Figure 1:**
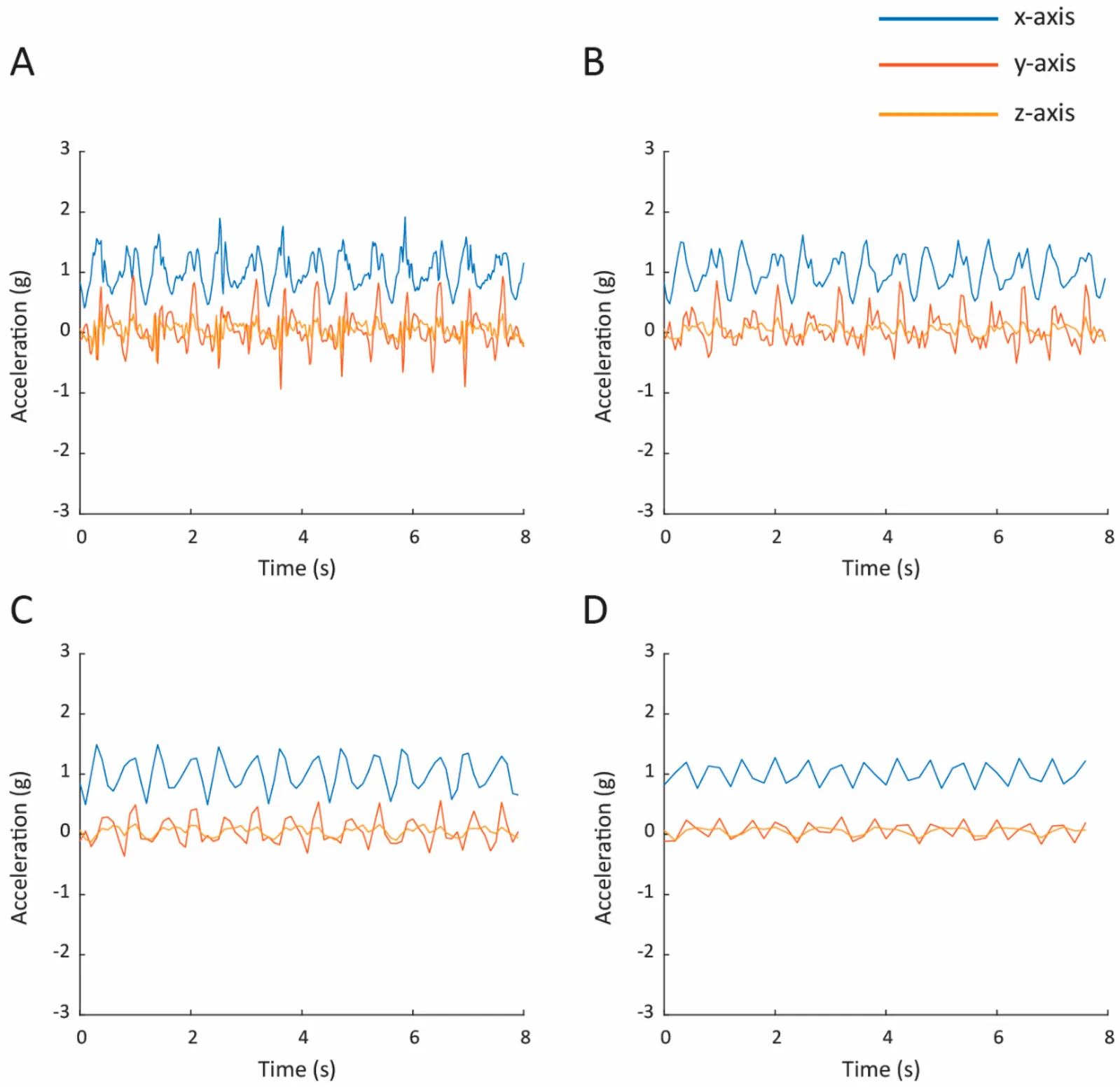
Exemplary accelerometer data of flat walking sampled at 50 (panel (**A**)), 20 (**B**), 10 (**C**), and 5 (**D**) Hz. Data were collected from the trunk of a *DaLiAc* study participant [10].

Analyses were restricted to datasets with an original sampling frequency of at least 50 Hz. For recordings containing timestamps, observations with non-finite acceleration or time values were removed, timestamps were ordered and duplicates removed, and the effective sampling frequency was calculated as the inverse of the median inter-sample interval. Signals recorded above 50 Hz were first converted to a standardized 50 Hz reference by resampling each acceleration axis independently utilizing rational polyphase rate conversion. Prior to decimation, a Kaiser-windowed linear-phase finite impulse response low-pass filter was applied to suppress frequency components exceeding the target Nyquist frequency, while compensating for filter group delay. Signals investigated at lower frequencies (40, 30, 20, 10, and 5 Hz) were systematically derived from these standardized 50 Hz reference signals using the identical downsampling procedure (Figure 1).

### 2.3. Physical Activity Metrics

To evaluate the impact of sampling frequency on physical activity intensity estimations, we extracted three established digital metrics of physical activity intensity: MIMS, ENMO, and AI.

These metrics were selected because their calculations depend on different properties of the acceleration signal and therefore provide complementary measures for evaluating sampling-frequency effects [19]. In our evaluation, these metrics were calculated separately from the standardized reference signals and the signals downsampled to 40, 30, 20, 10, and 5 Hz. Downsampling was expected to affect these measures through different mechanisms. Anti-aliased resampling attenuates signal components above the target Nyquist frequency and may smooth short-duration acceleration changes. For MIMS, this may reduce acceleration subsequently retained by the filtering and integration procedure [6]. ENMO may be affected by attenuation of transient changes in vector magnitude [7], whereas AI may decrease when higher-frequency within-second variability is removed [8]. The magnitude of these effects was expected to depend on both the underlying activity and sensor location.

#### 2.3.1. Monitor-Independent Movement Summary (MIMS)

MIMS was calculated using the processing steps proposed by John et al. (2019) [6]. For each candidate sampling frequency, the three acceleration axes were linearly reconstructed on a 100 Hz working grid. A fourth-order Butterworth band-pass filter with cut-off frequencies of 0.2 and 5 Hz was then applied to each axis in the forward and reverse directions to avoid phase displacement. The filtered signals were rectified, and the area under each rectified signal was calculated within each epoch using trapezoidal integration. For an epoch containing N  samples on the 100 Hz grid, a positive axis-specific integral not exceeding 0.0001 N  was set to zero to suppress small residual values. The retained integrals from the three axes were subsequently summed. Values from partial epochs were multiplied by 60/d, where d denotes the epoch duration in seconds. Results were expressed as MIMS units per standardized 60 s epoch (MIMS/min). The published MIMS algorithm additionally provides a spline-based procedure for reconstructing observations affected by sensor saturation. This procedure was not applied because device-specific clipping limits were not consistently available across the source datasets. Consequently, the estimates should be interpreted as MIMS values calculated without clipping extrapolation.

#### 2.3.2. Euclidean Norm Minus One (ENMO)

ENMO was calculated from tri-axial acceleration (x, y,z) expressed in gravitational units following the method of van Hees et al. (2013) [7]. For sample i, vector magnitude was defined as

(1) $$VM_{i}=\sqrt{x_{i}^{2}+y_{i}^{2}+z_{i}^{2}}$$

and the corresponding ENMO value was calculated as

(2) $$ENMO_{i}=max(VM_{i}-1,0)$$

Thus, the nominal contribution of gravity was subtracted from the vector magnitude, and negative values were truncated at zero. Sample-level values were averaged within each epoch and multiplied by 1000 to express ENMO in milligravitational units (mg). No additional offset or scale autocalibration was applied; residual differences in calibration among devices, therefore, remained in the estimates.

#### 2.3.3. Activity Index (AI)

AI was calculated at a one-second resolution and subsequently aggregated to 60 s epochs following the method of Bai et al. (2016) [8]. Within second s, the sample variance was calculated separately for each acceleration axis. The second-level AI was defined as

$$AI_{s}=\sqrt{\frac{1}{3}\sum_{j=1}^{3}\max\left\{Var,\left(a_{j,s}\right)-\sigma_{noise,j}^{2}0\right\}},$$

where $a_{j,s}$ denotes the samples from axis j during second s, and $\sigma_{noise,j}$ denotes the corresponding device-noise standard deviation. Minute-level AI was obtained by summing the available second-level values, equivalently multiplying their mean by 60 after normalization of a partial epoch. Device-specific stationary recordings were not consistently available; therefore, $\sigma_{noise,j}$ was set to zero for all axes. The resulting measure represents uncorrected within-second acceleration variability and is reported in AI units per standardized 60 s epoch.

### 2.4. Frequency Characteristics

Frequency-domain characteristics were estimated independently at each evaluated sampling frequency. Before spectral analysis, the mean was removed separately from each acceleration axis. Power spectral density was estimated for each axis using Welch’s method, with an 8 s periodic Hann window, 50% overlap, and a Fast Fourier Transform length equal to the larger of 256 samples and the next power of two above the window length. For activity bouts shorter than 8 s, the complete available signal was used as the analysis window. The three axis-specific power spectral densities were summed to obtain a single spectrum for each measurement.

### 2.5. Statistical Analysis

Five analyses were conducted to evaluate the effects of reduced sampling frequency on accelerometer signals and derived physical activity estimates. These included the assessment of dominant spectral peak location, retained spectral power, estimates of three physical activity metrics (MIMS, ENMO, and AI), and relative estimation error with respect to the 50 Hz reference. Additionally, we examined whether estimation errors were associated with participant age, sex/gender, and BMI.

The predominant movement frequency was characterized by identifying the dominant spectral peak location, defined as the frequency corresponding to the greatest spectral power within a prespecified frequency range.

Spectral retention was quantified by calculating the proportion of the 50 Hz reference spectral power located below the Nyquist frequency of each candidate sampling rate. This retained spectral power proportion ($R_{f_{s}}$) was calculated using the following formula:

(3) $$R_{f_{s}}=\frac{\int_{0}^{f_{s}/2}P_{ref}\left(f\right)df}{\int_{0}^{f_{max}}P_{ref}\left(f\right)df}.$$

To quantify the deviation between intensity metrics derived at downsampled frequencies ($M_{f_{s}}$) and the 50 Hz high-frequency reference ($M_{ref}$), the relative estimation error was calculated as

(4) $$E_{M,f_{s}}=\frac{\left|M_{f_{s}}-M_{ref}\right|}{\left|M_{ref}\right|}.$$

An a priori margin of equivalence was defined at an acceptable relative error threshold of <0.05 (<5%). A sampling frequency was deemed sufficient if the 95% confidence interval of the paired estimation error fell entirely within this predefined margin.

To determine whether errors introduced by downsampling were disproportionately associated with participant demographics, we further evaluated the relationship between estimation error and participant age, sex/gender, and Body Mass Index (BMI) using linear mixed-effects models (LMMs) to account for repeated within-subject observations. Estimation error was specified as the dependent variable in separate analyses for the MIMS, ENMO, and AI metrics across relative error scales, while excluding the 50 Hz reference standard observations, which possessed an error of zero, by definition. Each model included a participant-specific random intercept alongside fixed effects for categorical sex, standardized age, and BMI (calculated by subtracting the sample mean and dividing by the standard deviation), while adjusting for sampling frequency, activity type, sensor location, and dataset. The resulting coefficients are reported with 95% confidence intervals.

Statistical summaries throughout the analysis utilize the mean and standard deviation for normally distributed variables, while medians and interquartile ranges are employed, where appropriate. All calculations were performed using MATLAB 2024b (Mathworks, Natick, MA, USA).

## 3. Results

### 3.1. Data Summary

The investigation was conducted using data from 321 subjects in 11 publicly available datasets. Cumulatively, our analysis included over 315.4 h of accelerometer measurements split into 5676 bouts, with more than 85.2 h of data representing flat walking, 43.4 h of stairs climbing, 28.0 h of running, 21.7 h of cycling, 5.6 h of jumping, 86.4 h of stationary activities, 35.5 h of motorized transportation, and 9.8 h of household activities (Table 2). A detailed dataset summary is provided in Supplementary Materials, Table S1.

### 3.2. Effect of Sampling Rate on Spectral Characteristics

#### 3.2.1. Dominant Peak Location

**Figure 2:**
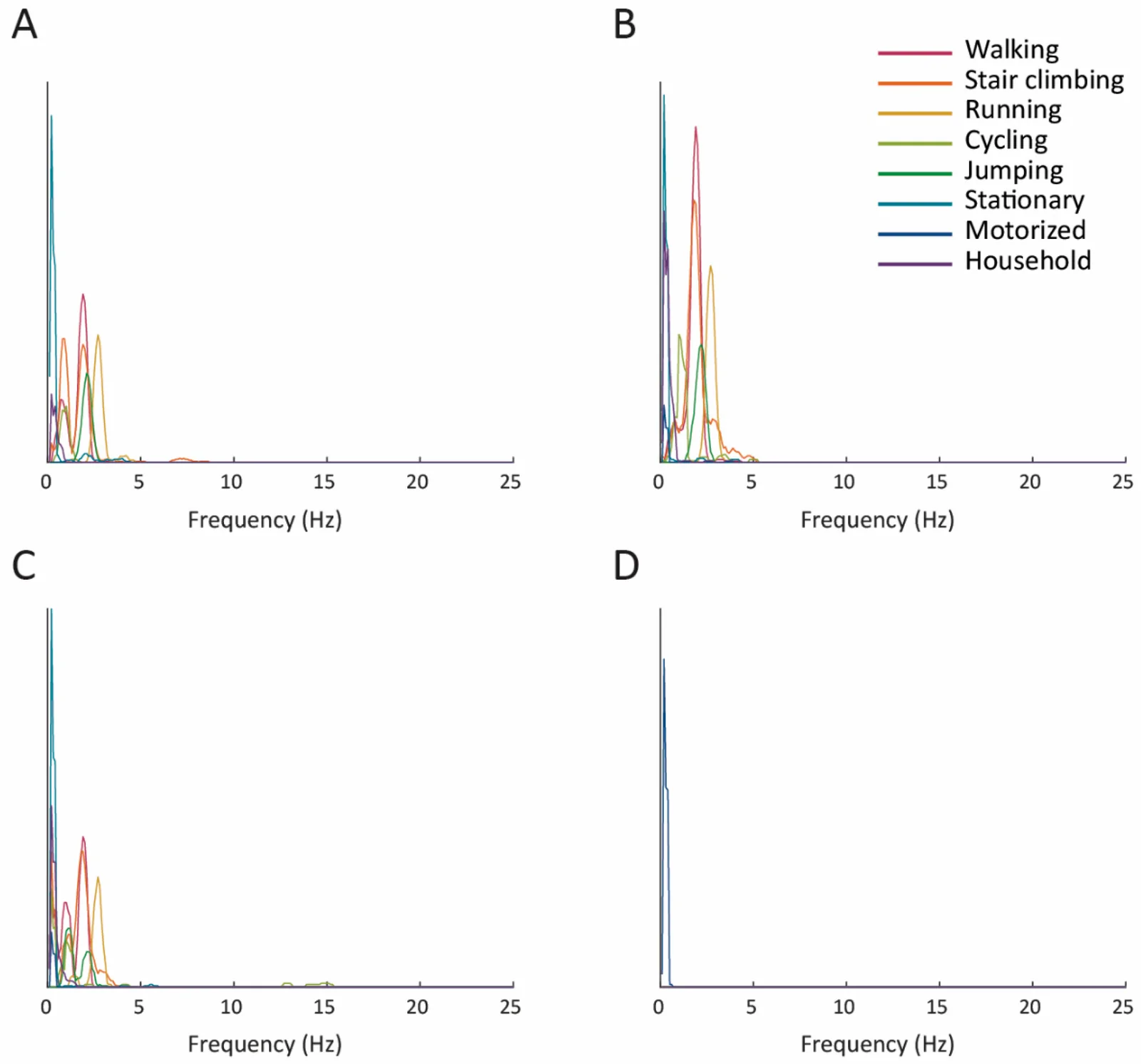
Dominant spectral peak location across accelerometer sampling frequencies. The dominant peak was defined as the frequency with the greatest spectral power within the prespecified frequency range. Results are stratified by sensor location: (**A**) lower limb, (**B**) trunk, (**C**) upper limb, and (**D**) unconstrained placement. Colors distinguish physical activity types. Stability across sampling frequencies indicates preservation of the predominant movement frequency.

Across the evaluated sensor body locations, ambulatory and locomotor activities exhibited distinct, identifiable dominant spectral frequencies (Figure 2). Running consistently resulted in the highest fundamental movement frequency, demonstrating consistency across investigated body regions: lower limb (mean ± SD: 2.71 ± 0.40 Hz), trunk (2.66 ± 0.17 Hz), and upper limb (2.53 ± 0.39 Hz). Similarly, jumping was characterized by high-frequency peaks centered around 2.06 ± 0.20 Hz at the lower limb and 2.09 ± 0.23 Hz at the trunk. Rhythmic locomotor tasks, including flat walking and stair climbing, generated dominant peaks primarily between 1.20 and 1.90 Hz; walking yielded dominant frequencies of 1.52 ± 0.54 Hz at the lower limb and 1.71 ± 0.44 Hz at the trunk, whereas stair climbing peaked at 1.57 ± 1.13 Hz and 1.93 ± 0.75 Hz, respectively. Cycling exhibited lower predominant frequencies at the lower limb (0.88 ± 0.20 Hz) and trunk (1.28 ± 0.78 Hz).

In contrast, non-rhythmic and low-intensity behaviors concentrated spectral power almost exclusively in the lowest frequency bins across all placement configurations. Stationary postures produced dominant peaks below 0.35 Hz (lower limb, 0.32 ± 0.70 Hz; trunk, 0.20 ± 0.48 Hz; upper limb, 0.22 ± 0.62 Hz). Similarly, motorized transportation modes evaluated across the trunk, upper limb, and unconstrained placements were characterized by low dominant frequencies with minimal variation, remaining near 0.11–0.13 Hz (unconstrained placement 0.13 ± 0.04 Hz). Household activities followed a comparable low-frequency profile, with mean dominant frequencies ranging between 0.28 Hz and 0.30 Hz across investigated regions.

Although the predominant movement frequencies were generally preserved at lower sampling rates, identification of the dominant spectral peak does not indicate how much of the remaining spectral content is retained. We therefore examined the proportion of reference spectral power available at each candidate sampling frequency.

#### 3.2.2. Retained Spectral Power

**Figure 3:**
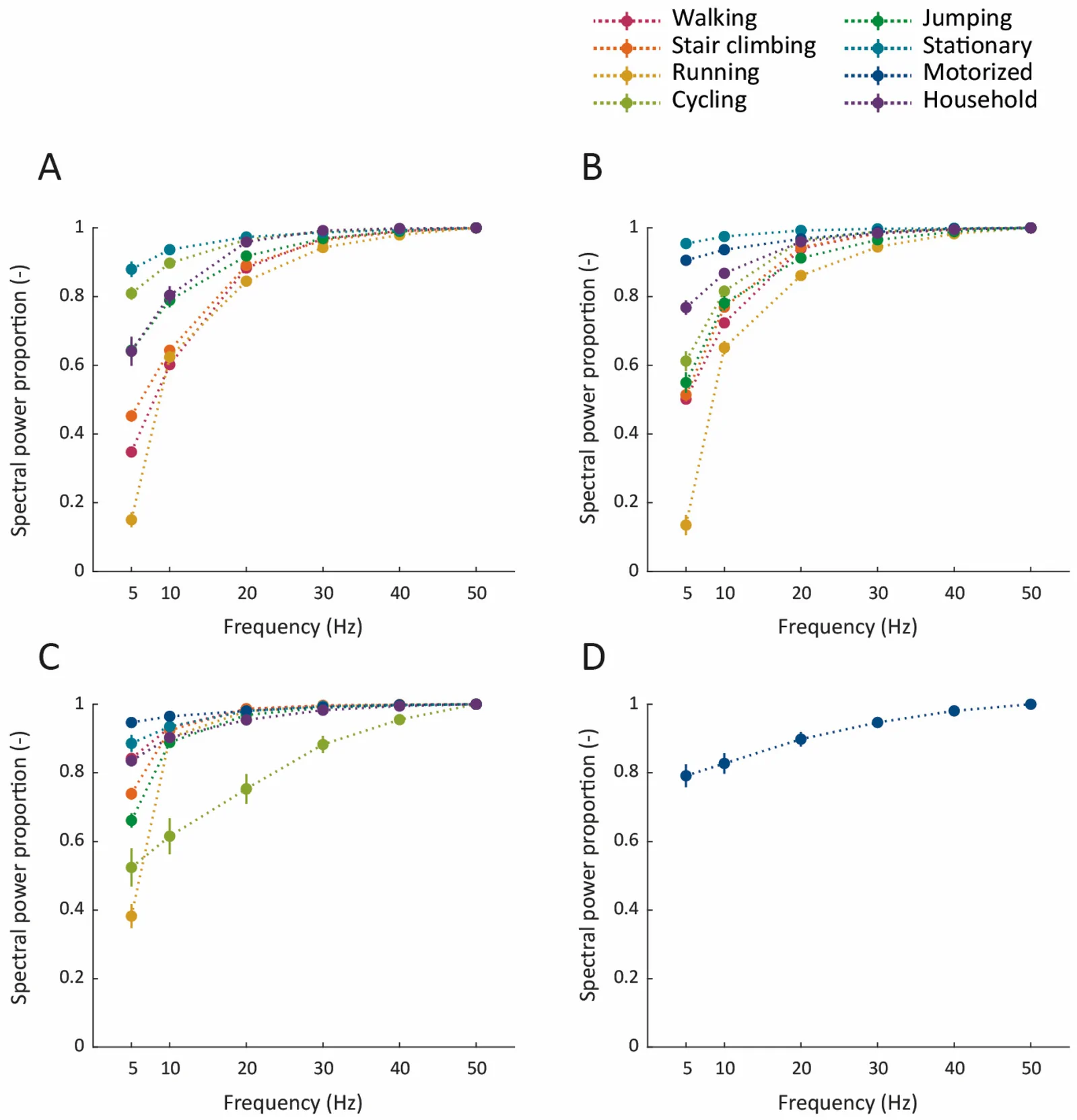
Retained spectral power across accelerometer sampling frequencies. Retained spectral power was defined as the proportion of the 50 Hz reference spectral power below the Nyquist frequency of each candidate sampling rate. Results are stratified by sensor location: (**A**) lower limb, (**B**) trunk, (**C**) upper limb, and (**D**) unconstrained placement. Dots indicate mean values, while solid vertical lines indicate 95% CI. Colors distinguish physical activity types. Values closer to 1 indicate greater preservation of the reference signal’s spectral content.

To verify the proportion of spectral power retained at lower sampling rates, 50 Hz reference signals were progressively downsampled to 5 Hz (Figure 3). Downsampling to 40 and 30 Hz resulted in near-complete preservation of signal characteristics across all physical activities and sensor placements, with retained spectral power proportions consistently exceeding 0.94. Even for vigorous tasks at 30 Hz, retention remained high, as observed for running (lower limb: 0.943 [95% CI: 0.936, 0.949]; trunk: 0.944 [0.939, 0.950]) and stair climbing (lower limb: 0.964 [0.961, 0.967]; trunk: 0.983 [0.982, 0.984]). Further reduction to 20 Hz maintained acceptable spectral retention, exceeding 0.84 across all activities, including 0.883 [0.874, 0.892] for walking and 0.845 [0.833, 0.857] for running, using lower limb sampling. This suggests that sampling frequencies between 20 Hz and 30 Hz might offer a physiological threshold for human activity recognition applications, effectively capturing the kinetic energy of ambulatory movements while minimizing data storage and battery consumption requirements, as compared to the results for higher sampling rates.

However, downsampling below 20 Hz substantially degraded the retained spectral power for dynamic ambulatory activities, indicating a loss of high-frequency biomechanical features which might be needed for movement quantification. At 10 Hz, the retained proportion for running dropped to 0.624 [0.607, 0.641] on the lower limb and 0.651 [0.632, 0.670] on the trunk, while for walking, it decreased to 0.602 [0.588, 0.616] and 0.724 [0.713, 0.734], respectively. The greater loss of spectral power during running and other dynamic activities was also consistent with our expectation that signals containing more higher-frequency content would be more sensitive to downsampling, which gradually removed information from higher harmonics (although retaining their fundamental harmonic, as presented in Figure 2).

At the minimum evaluated rate of 5 Hz (Nyquist frequency = 2.5 Hz), high-intensity activities experienced severe power attenuation due to dominant movement components exceeding the Nyquist threshold, rendering such low frequencies inadequate for analyzing vigorous physical behaviors. Running retained only 0.150 [0.129, 0.172] of the 50 Hz reference power at the lower limb, 0.135 [0.106, 0.165] at the trunk, and 0.382 [0.347, 0.417] at the upper limb. Walking exhibited a similar pronounced power loss at 5 Hz, retaining only 0.348 [0.333, 0.362] of its reference spectral power on the lower limb and 0.501 [0.490, 0.513] on the trunk. In contrast, low-frequency behaviors were largely unaffected by aggressive downsampling; at 5 Hz, stationary activities maintained 0.879 [0.857, 0.902] of spectral power at the lower limb and 0.954 [0.941, 0.968] at the trunk, while motorized transportation retained 0.905 [0.893, 0.918] at the trunk and 0.791 [0.758, 0.825] in unconstrained placements. These results indicate that spectral-power retention at 5 Hz was greater for stationary and motorized-transportation conditions than for ambulatory activities. These results also indicate that preservation of the predominant movement frequency can coexist with substantial loss of broader spectral content. We next examined whether this frequency-dependent attenuation was reflected in the accelerometry-derived movement measures.

### 3.3. Impact of Sampling Rate on Physical Activity Estimates

**Figure 4:**
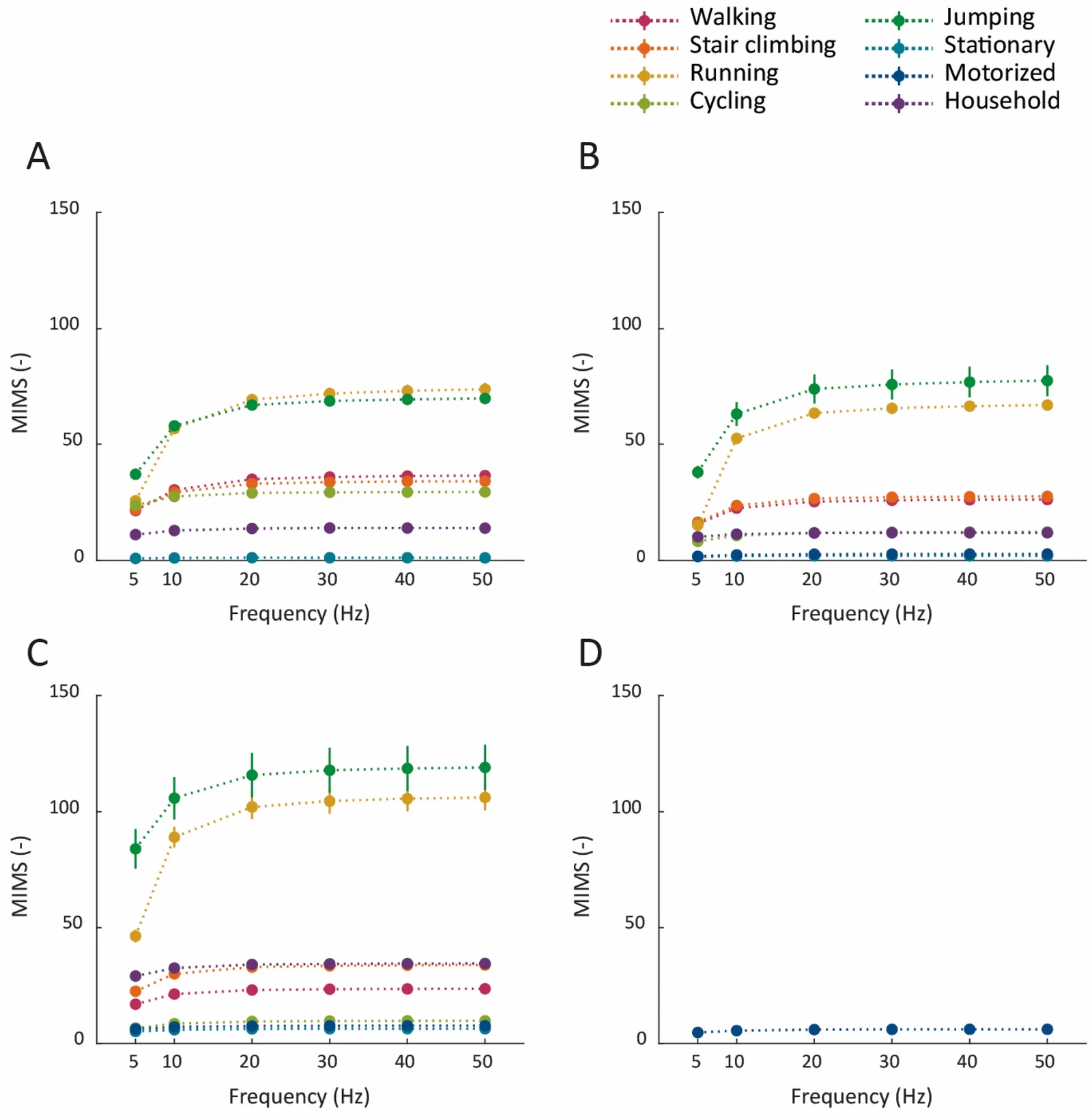
Monitor-Independent Movement Summary (MIMS) estimates across accelerometer sampling frequencies. MIMS estimates are shown by sensor location: (**A**) lower limb, (**B**) trunk, (**C**) upper limb, and (**D**) unconstrained placement. Dots indicate mean values, while solid vertical lines indicate 95% CI. Colors distinguish physical activity types. Variation across sampling frequencies indicates the extent to which sampling rate influences estimated movement intensity.

The estimated movement intensity, measured via MIMS, was highly sensitive to accelerometer sampling rates during vigorous physical activities. Running exhibited the most drastic MIMS underestimation at lower sampling frequencies across all sensor placements; on the lower limb, running MIMS dropped from 73.6 [95% CI: 71.0, 76.3] at 50 Hz to 56.7 [54.7, 58.7] at 10 Hz and plummeted to 25.7 [24.4, 27.1] at 5 Hz (Figure 4). Similar severe attenuation for running occurred at the trunk (66.8 [64.5, 69.1] at 50 Hz vs. 15.3 [14.1, 16.4] at 5 Hz) and the upper limb (106.0 [100.7, 111.4] at 50 Hz vs. 46.3 [43.7, 49.0] at 5 Hz). Moderate-intensity activities like flat walking also suffered substantial signal loss at 5 Hz, with lower limb MIMS falling to 21.5 [21.0, 22.0] compared to the 50 Hz reference of 36.5 [35.3, 37.7]. Conversely, low-intensity and sedentary tasks demonstrated relative stability across frequencies; household activities via lower-limb sensing yielded an MIMS of 13.9 [12.9, 15.0] at 50 Hz and 11.2 [10.4, 12.0] at 5 Hz, while unconstrained motorized transport varied minimally between 6.1 [5.7, 6.5] and 4.7 [4.4, 5.1]. This demonstrates that sampling rate reductions disproportionately truncate the high-amplitude, high-frequency acceleration spikes characteristic of vigorous movements, leading to a profound underestimation of actual energy expenditure.

ENMO and AI estimates also generally decreased with reductions in sampling frequency, although the magnitude of these differences varied between metrics, activities, and sensor locations (Supplementary Figures S1 and S2). For both metrics, the most pronounced reductions were observed during running, particularly for accelerometers placed on the lower limb and trunk. In contrast, estimates for walking, stair climbing, and several lower-intensity activities showed smaller absolute differences. Differences between metrics were also apparent for upper-limb measurements. For example, jumping estimates were comparatively well preserved at lower sampling frequencies, whereas cycling exhibited greater sensitivity to downsampling. These observations indicate that the effects of sampling frequency were not determined by activity type or movement intensity only.

Although these comparisons demonstrated sampling-frequency-dependent differences in metric estimates, they did not establish whether the observed discrepancies were within an acceptable error margin. We therefore evaluated relative estimation errors against the 50 Hz reference using the prespecified 5% criterion.

### 3.4. Error in Estimating Metrics

**Figure 5:**
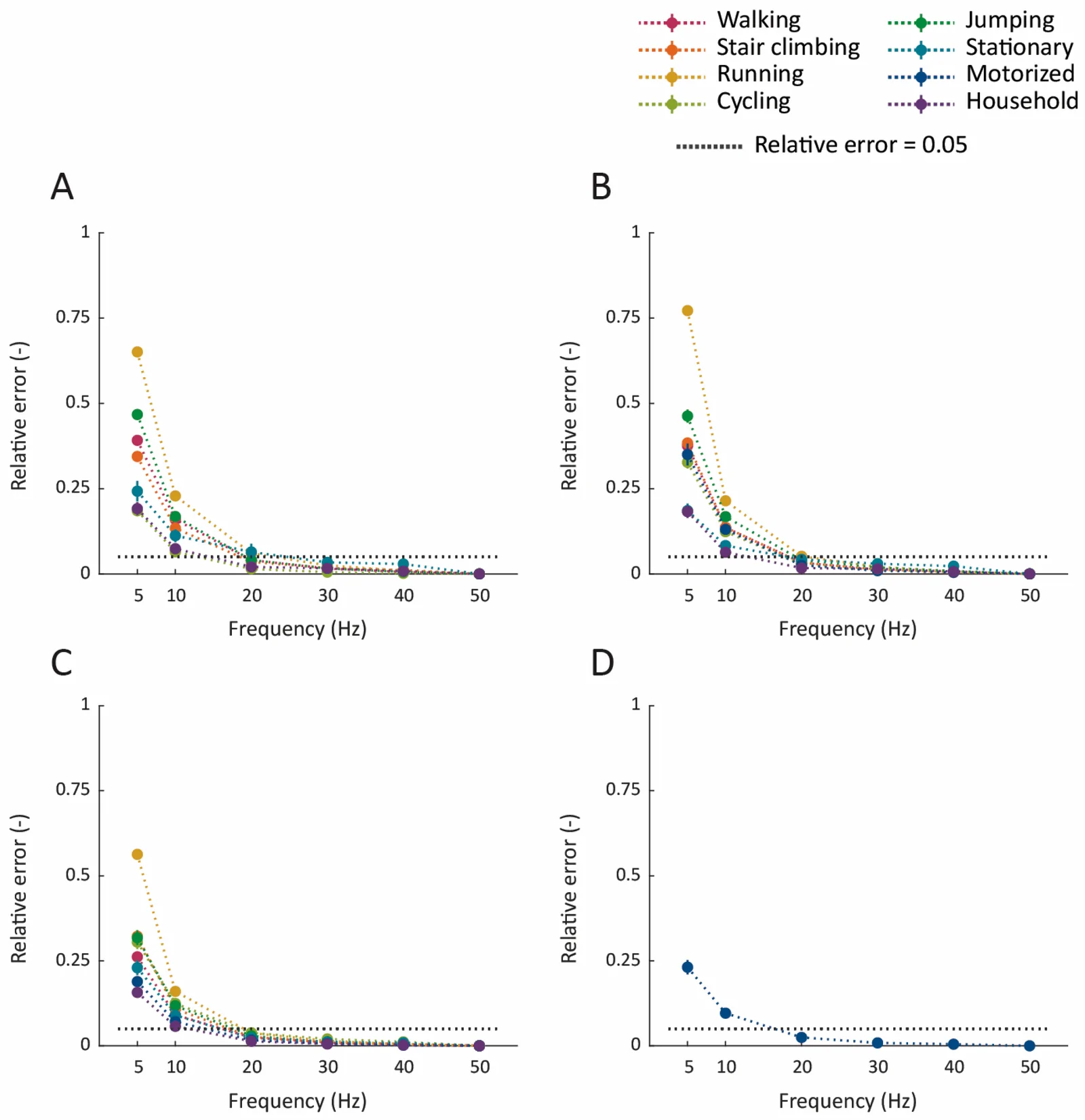
Relative error in Monitor-Independent Movement Summary (MIMS) estimates across accelerometer sampling frequencies. Relative error was calculated against the corresponding 50 Hz estimates. Results are stratified by sensor location: (**A**) lower limb, (**B**) trunk, (**C**) upper limb, and (**D**) unconstrained placement. Dots indicate mean values, while solid vertical lines indicate 95% CI. Colors distinguish physical activity types. Lower values indicate closer agreement with the 50 Hz reference. The dotted black horizontal line indicates 0.05 error criterion.

Relative error analysis revealed specific sampling frequency thresholds in which movement intensity estimates deviated from the 50 Hz reference (Figure 5). Highly dynamic tasks breached the cutoff of 0.05 at relatively high sampling rates, e.g., running activity downsampled to 20 Hz on both the lower limb (error: 0.060 [95% CI: 0.058, 0.062]) and trunk (0.052 [0.050, 0.053]). Stationary activities also exhibited threshold failures at higher frequencies, failing at 30 Hz on the lower limb (0.034 [0.018, 0.050]) and 20 Hz on the trunk (0.043 [0.034, 0.051]), possibly because their inherently low absolute MIMS values mathematically amplified minor discrepancies into large relative errors. Most other ambulatory and repetitive tasks, including walking, stair climbing, jumping, and cycling, maintained acceptable error margins at 20 Hz, but consistently failed when downsampled to 10 Hz across all sensor placements. For example, flat walking monitored on the lower limb exhibited a relative error of 0.040 [0.039, 0.042] at 20 Hz, but spiked to a 0.160 [0.155, 0.164] at 10 Hz. For household activities and motorized transportation, we observed acceptable estimates at 20 Hz which exceed the cutoff when downsampled to 10 Hz (e.g., trunk motorized transport error reached 0.131 [0.114, 0.147] at 10 Hz). Consequently, while 20 Hz emerges as the minimum sampling frequency required to capture reliable intensity estimates for general everyday behaviors, a sampling rate of 30 Hz or higher appears necessary for studies aiming to accurately quantify vigorous physical activities.

Relative estimation errors further demonstrated differences between MIMS, ENMO, and AI (Supplementary Figures S3 and S4). At 20 Hz, the ENMO relative error was approximately 0.10 for lower-limb running and 0.23 for upper-limb cycling, exceeding the prespecified 0.05 criterion. Corresponding AI errors were smaller, approximately 0.05 and 0.16, respectively. Particularly pronounced differences were observed during unconstrained motorized transportation, for which ENMO relative error remained approximately 0.37 at 20 Hz and 0.07 at 40 Hz, compared with approximately 0.20 and 0.04 for AI. Thus, although all three metrics showed increasing discrepancies with decreasing sampling frequency, conditions meeting the error criterion for MIMS did not necessarily meet the same criterion for ENMO or AI. These findings suggest that sampling-frequency requirements should be established separately for each accelerometry-derived metric rather than inferred from the behavior of another measure.

Having established that estimation errors varied across metrics, activities, and sensor locations, we next examined whether these errors were also associated with participant demographic and anthropometric characteristics.

### 3.5. Bias Estimation

Linear models evaluated the associations between participant demographic characteristics (age, sex/gender, and BMI) and the relative estimation error across physical activity metrics (Table 3). For the primary metric (MIMS), relative error was not significantly associated with age, source-reported sex/gender, or BMI. These results provided no evidence of systematic associations with the investigated participant characteristics in the available sample.

In contrast, two other physical activity estimates exhibited a small association with source-reported demographic covariates. For the ENMO metric, male sex/gender was associated with a statistically significant increase in relative estimation error (0.0110 [95% CI: 0.0055, 0.0164]), whereas age and BMI did not significantly influence the error margins (in both cases, confidence intervals included zero). Similarly, relative error in the AI metric demonstrated a statistically significant association with sex/gender (0.0063 [95% CI: 0.0002, 0.0105]), while remaining unassociated with age and BMI.

## 4. Discussion

### 4.1. Principal Findings

Our findings suggest that there is unlikely to be a single accelerometer sampling frequency that is sufficient for all physical activity measurements. After harmonizing raw tri-axial acceleration from 11 independent datasets to a common 50 Hz reference and progressively reducing the sampling frequency using anti-aliased polyphase resampling, the magnitude of the resulting error varied according to activity, sensor location, and physical activity metric. For MIMS, 20 Hz was sufficient under the prespecified 5% error criterion for walking, stair-climbing, cycling, jumping, household, and motorized-transportation conditions, whereas for running, the error exceeded this margin at 20 Hz at both the lower-limb and trunk locations. Reducing the sampling frequency to 10 Hz resulted in larger discrepancies for most ambulatory activities. Similar patterns were observed for ENMO and AI.

The frequency-domain results help explain these observations. The dominant frequency of flat walking was approximately 1.5–1.7 Hz and that of running approximately 2.5–2.7 Hz, values that could theoretically be represented at relatively modest sampling frequencies. Nevertheless, substantial signal power occurred above these fundamental frequencies. For example, at 10 Hz, approximately 60% of the 50 Hz spectral power was retained for lower-limb walking and 62% for lower-limb running. Thus, satisfying the Nyquist criterion for the dominant movement rhythm does not necessarily preserve the signal components contributing to movement-intensity summaries. This distinction is also consistent with our previous observation that walking comprises subharmonic and higher-harmonic components whose relative importance varies with sensor location and gait characteristics [20].

### 4.2. Findings in the Context of Previous Work

Previous investigations have similarly suggested that the effect of sampling frequency depends on the analytical outcome. Brønd and Arvidsson showed that ActiGraph activity counts varied with acquisition frequency because sampling frequency interacted with the device-processing filter, with particularly large differences during running [1]. In children, Clevenger et al. found relatively small effects of reducing 100 Hz data to 30 Hz on raw acceleration but clearer effects on activity counts, with differences increasing with activity intensity [2]. Subsequent free-living observations in 445 children and adolescents found MIMS, mean amplitude deviation, and several activity-intensity estimates to be statistically equivalent between 30 and 100 Hz. These studies are compatible with the present observations, i.e., that increasing sampling frequency substantially above 30 Hz may provide limited additional information for some physical activity summaries, whereas reductions below this range can become increasingly consequential.

Importantly, digitally downsampling a higher-frequency recording should not be assumed to fully reproduce data acquired natively at the lower rate. Small et al. compared simultaneously recorded 25 and 100 Hz accelerometry in healthy adults and found strong correlations between measurements, but approximately 12–13% lower overall acceleration at 25 Hz [3]. Their secondary downsampling of the 100 Hz recording did not reproduce the same difference, suggesting contributions from device-level acquisition and processing. This is particularly relevant to the present study, in which downsampling isolates the effect of removing higher-frequency information under a standardized signal-processing procedure but cannot reproduce differences in analogue filters, sensor electronics, firmware, or noise characteristics.

Lower sampling frequencies may nevertheless be entirely appropriate for specific algorithms. Zhang et al. reported similar classification accuracy at 10–80 Hz for recognition of sedentary, household, walking, and running activities in 60 adults, with performance decreasing principally at 5 Hz [21]. Our previous walking-recognition method similarly standardized heterogeneous accelerometer data to 10 Hz and retained high sensitivity and specificity across 20 datasets and multiple sensor locations [20]. A related smartphone step-counting algorithm used 10 Hz data and was evaluated in both nonclinical cohorts and individuals with advanced cancer [22]. These findings do not conflict with the present results, as identifying a periodic event or quantifying walking does not require preservation of all of the acceleration magnitude or spectral power contained in the original signal.

Evidence from clinical populations further argues against a universal threshold. Ghaffari et al. found that accelerometers operating at approximately 12.5 Hz could provide useful time- and frequency-domain measurements of overground walking in older individuals with knee osteoarthritis [23]. Conversely, Shawen et al. found that sampling frequency affected wearable-based recognition of Parkinsonian tremor, while bradykinesia quantification was considerably less sensitive to sampling rate [24]. In contrast, Aubol and Milner recommended approximately 199 Hz when the target outcome was peak tibial acceleration during running [25], which suggested that the required sampling rate is determined at least as much by the target digital measure as by the population being studied.

Motorized transportation illustrates this point well. In our data, its dominant frequency was close to 0.1 Hz, and much of its total spectral power was retained at low sampling frequencies. However, trunk MIMS at 10 Hz differed from the 50 Hz reference by approximately 13%, indicating that low dominant frequency alone was not sufficient to ensure preservation of an intensity metric. Moreover, previous work on accelerometer-based driving recognition exploited vehicle-related vibrations between approximately 10 and 16 Hz [26]. A low sampling frequency might therefore be adequate for one transportation-related feature, while eliminating information required by another [27]. Combining accelerometry with GPS may reduce dependence on high-frequency acceleration for transportation estimation, but this was not evaluated in the present analysis [28].

### 4.3. Implications for Study Design

These findings support a fit-for-purpose approach to accelerometer data acquisition. Sampling at 50 rather than 10 Hz produces five times as many acceleration samples, with corresponding increases in raw data volume and downstream storage and processing requirements. Lower rates may also reduce power consumption, although the magnitude of this benefit is hardware- and software-dependent rather than proportional to sampling frequency. Our previous systematic review found that smartphone HAR studies most commonly sampled inertial sensors at approximately 20–30 Hz and identified examples in which reducing sampling frequency substantially prolonged battery life [28].

The practical importance of these activity-specific differences may also depend on the behavioral composition of the population being studied. Free-living adults generally spend substantially more time in stationary and low-intensity behaviors than in highly dynamic activities. For example, 24 h accelerometry in 1670 participants from the Copenhagen City Heart Study showed median values of 82.6 min/day of walking, 4.4 min/day of stair climbing, and only 0.1 min/day of running, compared with 579.1 min/day of sedentary behavior and 479.5 min/day in bed; among participants who cycled, the median cycling duration was 8.3 min/day [29]. Population averages, however, conceal considerable between-person heterogeneity. In a nationally representative accelerometry sample of 7931 US adults, Evenson et al. identified activity profiles ranging from approximately 9 min/day of moderate-to-vigorous physical activity in the least-active class to 114 min/day in the most-active class, while sedentary time ranged from approximately 256 to 660 min/day across corresponding behavioral patterns [30]. Other daily activities can also occupy substantial portions of the day; for example, older adults in the OUTDOOR ACTIVE study reported approximately 80–119 min/day of home-based activities, including household chores, gardening, and repairs [31]. Travel behavior may further alter the distribution of movement, with UK Biobank data indicating that participants who walked, cycled, or used mixed travel modes were generally less sedentary and more physically active than those relying on motorized transport [32].

These observations also suggest that the consequences of reducing sampling frequency may differ across study populations, even when the same accelerometry-derived metric is used. In predominantly sedentary populations, a large proportion of the monitored day may consist of behaviors that are relatively insensitive to reductions in sampling frequency, whereas more active populations may devote a greater proportion of monitored time to walking, running, cycling, and other dynamic activities for which larger downsampling-related differences were observed. This should not, however, be interpreted to mean that low-frequency behaviors universally permit low sampling rates: motorized transportation in the present analysis, for example, had a low predominant frequency but still showed metric-specific differences at reduced sampling frequencies. Sampling frequency should therefore be selected with consideration of the target metric, sensor location, expected distribution of free-living behaviors in the population, and acceptable level of measurement error.

This consideration may also be relevant to smartphone-based studies. Consumer-owned phones can differ in sensor hardware, operating-system restrictions, realized sampling frequency, missingness, and device location. A recent analysis of sensor data from 3000 smartphones documented measurable differences in sampling consistency and other data-quality properties between Android and iOS devices [33]. Accordingly, harmonization to a common sampling grid may facilitate analysis of heterogeneous smartphone data, but it cannot remove all device-specific differences.

### 4.4. Strengths and Limitations

A strength of the present analysis is that it included 321 participants from 11 datasets collected independently using smartphones, smartwatches, and research-grade accelerometers. The data covered multiple physical activities and four harmonized sensor-location categories, while each sampling-frequency comparison was derived from the same underlying signal. This design reduces variation that would arise if different participants or activity trials were compared between acquisition settings. Previous device-comparison studies suggest that selected raw and derived accelerometry measures can show good correspondence across devices when processed consistently, although differences in acceleration magnitude may remain [34,35].

Several limitations should be considered. Most measurements were obtained from young, healthy participants performing labelled activities under controlled conditions, and the broad activity categories used here cannot capture the full variability of free-living behavior. The demographic analysis provided no evidence that MIMS downsampling error was associated with demographic or anthropometric variables, while small associations with sex/gender were observed for ENMO and AI. These results should be interpreted as evidence about this study sample rather than as demonstration of demographic fairness, particularly given the limited representation of older and clinical populations. The analysis also evaluated preservation relative to the best available 50 Hz signal rather than physiological ground truth. Digital downsampling cannot reproduce all properties of native acquisition, and the results are specific to the applied anti-aliasing and metric implementations. Also, the present analysis used activity labels to identify known bouts and did not evaluate recognition or classification of activities in unlabeled free-living data. Accordingly, the sampling-frequency requirements reported here describe preservation of accelerometry-derived metrics and should not be interpreted as thresholds for human activity recognition performance [5]. Finally, we did not evaluate activity-specific outcomes, such as gait timing, cadence, stride variability, or impact acceleration. At 50 Hz, consecutive samples are separated by 0.02 s compared with 0.1 s at 10 Hz; this difference may be relatively unimportant for an epoch-level activity summary but consequential when individual gait events must be localized precisely.

Future work should extend these analyses to data acquired natively at different sampling frequencies because digital downsampling does not reproduce all differences associated with device-level acquisition and processing [3]. Further evaluation is also needed in older and clinical populations and during unconstrained free-living behavior. Sampling requirements should additionally be evaluated for outcome-specific measures, including gait-event timing, cadence, stride variability, and impact acceleration, as well as for activity-recognition algorithms developed for particular populations and sensing configurations. Finally, studies that directly quantify the effects of sampling frequency on battery use, data storage, and transmission would further inform acquisition protocols for longitudinal monitoring.

## 5. Conclusions

The principal implication of this study is that sampling frequency should be considered as part of the validation of an accelerometry-derived measure, rather than as a device setting selected independently of the intended outcome. While the greater sensitivity of dynamic activities to downsampling was consistent with our hypothesis, the observation that dominant movement frequency generally fell within the bandwidth available at lower sampling rates, despite substantial loss of broader spectral power, suggests that different accelerometry-derived outcomes may require different sampling frequencies. Across the investigated activities, 20 Hz often preserved commonly used movement-intensity summaries relative to 50 Hz, whereas higher sampling rates were required to meet the prespecified error criterion for some vigorous activities, including running. At 10 Hz, these measures generally exhibited greater estimation errors, although previous studies have demonstrated that this sampling frequency can be sufficient for specifically designed walking-recognition or step-counting algorithms. This distinction is important because algorithms designed to recognize or quantify specific behaviors may rely on signal features that remain detectable even when other frequency components are attenuated.

Therefore, sampling frequency requirements derived from activity-estimation performance should not be interpreted as requirements for preserving accelerometry-derived movement summaries, and vice versa. Instead, the sampling rate could be selected as the lowest rate shown to preserve the intended outcome within an acceptable error margin, considering sensor location, population, and practical requirements of longitudinal data collection [3,5,20,21]. This approach may provide a more useful basis for study design than the conventional selection of uniformly high sampling frequencies.

## Figures and Tables

**Table 1:** Summary of datasets included in this study. Age, height, weight, and BMI are provided as range (mean ± SD), when available. The terminology used for sex and gender varied among the source datasets. MR—measurement range, AR—amplitude resolution, and SR—approximate sampling rate.

		Investigated Activities	Population						Measurement Parameters						
Dataset Name	Dataset Acronym		N	Sex/Gender (Male)	Age (yr.)	Height (cm)	Weight (kg)	BMI (kg/m^2^)	Condition	Sensing Device	Approximate Sensor Location	MR (g)	AR (Bit)	SR (Hz)	Ref.
Complex Human Activity Dataset	*CHA*	Flat walking, ascending stairs, descending stairs, sitting, standing, typing, handwriting, eating, drinking, jogging, cycling	10	10	23 ÷ 35	N/A	N/A	N/A	Controlled	Smartphone: Samsung Galaxy S2	Thigh and wrist	N/A	N/A	50	[9]
Daily Life Activities Dataset	*DaLiAc*	Flat walking, ascending stairs, descending stairs, lying, sitting, standing, washing dishes, vacuuming, sweeping, running, cycling (50 W and 100 W), rope jumping	19	11	18 ÷ 55 (26.5 ± 7.7)	158 ÷ 196 (177.0 ± 11.1)	54 ÷ 108 (75.2 ± 14.2)	17 ÷ 34 (23.9 ± 3.7)	Controlled	Wearable accelerometer: SHIMMER	Waist, chest, and wrist	±6	12	204.8	[10]
Human Activities and Postural Transitions Dataset	*HAPT*	Flat walking, ascending stairs, descending stairs, standing, sitting, lying	30	N/A	19 ÷ 48	N/A	N/A	N/A	Controlled	Smartphone: Samsung Galaxy S2	Waist	N/A	N/A	50	[11]
Identification of Walking, Stair Climbing, and Driving using Wearable Accelerometers	*IWSDC*	Flat walking, ascending stairs, descending stairs, using motorized transportation: car	32	13	23 ÷ 52 (39.0 ± 9.0)	147 ÷ 193 (173.5 ± 11.1)	45 ÷ 140 (77.0 ± 22.9)	18 ÷ 40 (25.2 ± 5.6)	Controlled	Wearable accelerometer: ActiGraph GT3X+	Waist and wrist	N/A	N/A	100	[12]
Recognition of Activities of Daily Living using Smartphones	*MobiAct*	Flat walking, ascending stairs, descending stairs, lying, sitting, standing, jogging, jumping	61	42	20 ÷ 40 (24.9 ± 3.7)	158 ÷ 193 (175.9 ± 8.1)	50 ÷ 120 (76.8 ± 15.0)	18 ÷ 35 (24.7 ± 3.8)	Controlled	Smartphone: Samsung Galaxy S3	Thigh	N/A	N/A	200	[13]
Sensor-Based Human Activity and Attribute Recognition	*MotionSense*	Flat walking, ascending stairs, descending stairs, sitting, standing, jogging	24	14	18 ÷ 46 (28.8 ± 5.4)	161 ÷ 190 (174.2 ± 8.9)	48 ÷ 102 (72.1 ± 16.2)	18 ÷ 32 (23.6 ± 4.1)	Controlled	Smartphone: iPhone 6	Thigh	N/A	N/A	50	[14]
Physical Activity Recognition Dataset Using Smartphone Sensors	*PARDUSS*	Flat walking, ascending stairs, descending stairs, sitting, standing, jogging, cycling	10	10	25 ÷ 30	N/A	N/A	N/A	Controlled	Smartphone: Samsung Galaxy S2	Thigh, waist, arm, and wrist	N/A	N/A	50	[15]
Real-World Dataset	*RealWorld*	Flat walking, ascending stairs, descending stairs, lying, sitting, standing, running, jumping	15	8	16 ÷ 62 (31.9 ± 12.4)	163 ÷ 183 (173.1 ± 6.9)	48 ÷ 95 (74.1 ± 13.8)	18 ÷ 35 (24.7 ± 4.4)	Controlled	Smartphone: Samsung Galaxy S4, smartwatch: LG G Watch R	Smartphone: thigh, waist, chest, and arm; smartwatch: wrist	N/A	N/A	50	[16]
Speed-Breaker Dataset	*SpeedBreaker*	Using motorized transportation: car, motorcycle, and rickshaw	40	N/A	N/A	N/A	N/A	N/A	Free-living	Smartphone: Android-based	Unspecified	N/A	N/A	100	[17]
A Fall and Movement Dataset	*SisFall*	Flat walking: slow, fast; jogging: slow and fast; jumping,	38	19	19 ÷ 75 (40.2 ± 21.3)	149 ÷ 183 (164.1 ± 9.3)	41 ÷ 102 (62.2 ± 12.6)	18 ÷ 35 (23.0 ± 3.5)	Controlled	Wearable accelerometer: self-developed	Waist	±16	13	200	[18]
Human Physical Activity Dataset	*SPADES*	Flat walking, ascending stairs; descending stairs; treadmill walk at 1, 2, 3, and 3.5 mph; lying; sitting; standing; reclining; handwriting; typing; folding towels; filling shelves; sweeping; running; cycling; jumping jacks	42	27	18 ÷ 30 (23 ± 3)	151 ÷ 180 174 ± 8	51 ÷ 112 (73 ± 15)	18 ÷ 35 (24 ± 4)	Controlled	Wearable accelerometer: ActiGraph GT9X	Thigh, waist, and wrist	8	N/A	80	[6]

**Table 2:** Number of analyzed activity bouts and their total duration according to sensor location and activity type. In total, we investigated data from 5676 bouts over 315.4 h of measurement.

	Bouts, n	Duration, h
**Sensor location**		
Lower limb	2310	87.7
Trunk	2076	110.9
Upper limb	1211	102.2
Unconstrained	79	14.7
**Activity class**		
Flat walking	1008	85.2
Stair climbing	2047	43.4
Running	597	28.0
Cycling	304	21.7
Jumping	554	5.6
Stationary	673	86.4
Motorized transportation	133	35.5
Household activities	360	9.8
**Total**	**5676**	**315.4**

**Table 3:** Associations of age, sex/gender, and BMI with relative estimation error in physical activity metrics (MIMS, ENMO, and AI).

		Estimate	Standard Error (SE)	95% Confidence Intervals (95% CI)	*p*-Value
**MIMS**					
	Sex/gender (Male)	−0.0026	0.0015	(−0.0057, 0.0004)	0.085
	Age	−0.0021	0.0016	(−0.0052, 0.0010)	0.180
	BMI	0.0002	0.0015	(−0.0013, 0.0018)	0.732
**ENMO**					
	Sex/gender (Male)	0.0110	0.0028	(0.0055, 0.0164)	<0.001
	Age	−0.0048	0.0028	(−0.0104, 0.0008)	0.092
	BMI	−0.0001	0.0014	(−0.0029, 0.0028)	0.958
**AI**					
	Sex/gender (Male)	0.0063	0.0021	(0.0022, 0.0105)	0.002
	Age	−0.0033	0.0021	(−0.0075, 0.0008)	0.117
	BMI	0.0011	0.0011	(−0.0011, 0.0032)	0.323

## Data Availability

This study used data available in public repositories. Preprocessed data are available from the corresponding author upon reasonable request.

## Supplementary Materials

The following supporting information can be downloaded at https://www.mdpi.com/article/10.3390/s26206652/s1, Table S1. Number and duration of activity measurements by sensor-location region, dataset, and activity type; Table S2. Data collection protocols of the datasets included in the study. Figure S1: Euclidean Norm Minus One (ENMO) estimates across accelerometer sampling frequencies; Figure S2: Activity Index (AI) estimates across accelerometer sampling frequencies; Figure S3: Relative error in ENMO across accelerometer sampling frequencies; Figure S4: Relative error in AI across accelerometer sampling frequencies.

## Abbreviations

The following abbreviations are used in this manuscript:

MIMS	Monitor-Independent Movement Summary
ENMO	Euclidean Norm Minus One
AI	Activity Index
SD	Standard Deviation
