# Supplementary material for: Sampling Frequency Requirements for Estimating Physical Activity Metrics from Wearable Accelerometer Data
Source: Sensors (Basel). 2026 Oct 21;26(20):6652. doi: 10.3390/s26206652 (PMC13709298; doi:10.3390/s26206652)
Supplement: Supplementary file 1 [file sensors-26-06652-s001.zip › sensors-4567198-supplementary.pdf]

Supplementary materials

1.1 Dataset summary

Table S1. Number and duration of activity measurements by sensor-location region, dataset, and activity type. Duration is reported in minutes as the mean (standard deviation). Sensor locations were grouped into lower limb, trunk, upper limb, and unconstrained regions.

Sensor location	Dataset	Walking flat	Climbing stairs	Running	Cycling	Jumping	Stationary	Motorized	Household
**Lower limb**									
	CHA	10, 3 (0)	20, 3 (0)	10, 3 (0)	10, 3 (0)	-	50, 3.6 (1.21)	-	-
	MobiAct	61, 4.97 (0.01)	719, 0.122 (0.01)	183, 0.483 (0.00)	-	183, 0.486 (0.00)	61, 4.92 (0.51)	-	-
	MotionSense	72, 1.61 (0.60)	144, 0.681 (0.28)	48, 0.94 (0.55)	-	-	48, 2.15 (1.15)	-	-
	PARDUSS	20, 3 (0)	40, 3 (0)	20, 3 (0)	20, 3 (0)	-	20, 6 (0)	-	-
	RealWorld	15, 10.79 (0.41)	30, 8.08 (3.15)	15, 11.97 (2.65)	-	14, 1.63 (0.10)	15, 10.57 (0.36)	-	-
	SPADES	158, 5.79 (5.51)	75, 1.50 (2.59)	30, 2.24 (2.48)	40, 7.12 (4.10)	42, 0.551 (0.181)	74, 13.60 (10.15)	-	63, 1.67 (0.69)
**Trunk**									
	DaLiAc	38, 4.58 (1.27)	76, 0.66 (0.09)	38, 2.04 (0.02)	76, 2.07 (0.050)	34, 0.65 (0.23)	38, 3.06 (0.04)	-	114, 1.59 (0.51)
	HAPT	60, 0.67 (0.10)	120, 0.62 (0.07)	-	-	-	60, 2.21 (0.39)	-	-
	IUWDC	32, 8.21 (0.78)	64, 1.42 (0.20)	-	-	-	-	27, 23.09 (4.31)	-
	PARDUSS	10, 3 (0)	20, 3 (0)	10, 3 (0)	10, 3 (0)	-	10, 6 (0)	-	-
	RealWorld	30, 10.79 (0.40)	60, 8.08 (3.13)	30, 11.97 (2.60)	-	30, 1.63 (0.09)	30, 10.57 (0.36)	-	-
	SisFall	76, 1.69 (0.21)	308, 0.41 (0.00)	74, 1.70 (0.215)	-	120, 0.19 (0.01)	-	-	-
	SPADES	157, 5.75 (5.41)	75, 1.50 (2.59)	30, 2.24 (2.48)	40, 7.12 (4.10)	42, 0.55 (0.18)	74, 13.6 (10.15)	-	63, 1.67 (0.69)
**Upper limb**									

	CHA	10, 3 (0)	20, 3 (0)	10, 3 (0)	10, 3 (0)	-	50, 3.6 (1.21)	-	-
	DaLiAc	19, 4.58 (1.29)	38, 0.66 (0.09)	19, 2.04 (0.02)	38, 2.07 (0.05)	17, 0.65 (0.23)	19, 3.06 (0.04)	-	57, 1.59 (0.51)
	IUWDC	32, 8.21 (0.78)	64, 1.42 (0.20)	-	-	-	-	27, 23.09 (4.31)	-
	PARDUSS	20, 3 (0)	40, 3 (0)	20, 3 (0)	20, 3 (0)	-	20, 6 (0)	-	-
	RealWorld	30, 10.76 (0.40)	59, 8.02 (3.12)	30, 11.78 (2.84)	-	30, 1.62 (0.09)	30, 10.54 (0.36)	-	-
	SPADES	158, 5.72 (5.38)	75, 1.50 (2.59)	30, 2.24 (2.48)	40, 7.12 (4.10)	42, 0.55 (0.18)	74, 13.6 (10.15)	-	63, 1.67 (0.69)
**Unconstrained**									
	SB	-	-	-	-	-	-	79, 11.16 (8.78)	-

Table S2. Data collection protocols of the datasets included in the study.

Dataset	Data collection setting	Activity execution and participant instructions	Trial structure and duration	Activity labeling / ground truth
**CHA**	Predominantly controlled university environment. Most activities were performed indoors; cycling and smoking were performed outdoors.	Participants performed activities individually. Walking, jogging, cycling, stair ascent/descent, sitting, and standing were performed by all participants. Typing, handwriting, eating, drinking coffee, and giving a talk were performed by a subset. Sitting and standing were performed without concurrent activities; eating, drinking, writing, and typing followed activity-specific instructions.	Core locomotor and stationary activities were performed for approximately 3 min. Eating, typing, handwriting, drinking coffee, and giving a talk lasted approximately 5–6 min. Smoking consisted of one cigarette.	Activities were recorded using a dedicated Android data-collection application under researcher-defined experimental conditions.
**DaLiAc**	Mixed indoor and university-campus protocol. Static and household activities were performed indoors; walking and stairs involved movement between campus buildings; vigorous activities were subsequently performed indoors.	Participants first performed sitting, lying, standing, and household activities. They then walked across the university campus, ascended to the third floor and descended the stairs, ran on a treadmill, cycled on an ergometer, and performed rope jumping. Treadmill speed was fixed at 8.3 km/h; cycling was performed at 50 and 100 W while maintaining approximately 70 rpm.	Static and household tasks generally lasted 1–2 min. Treadmill running and each cycling condition lasted 2 min. Rope jumping consisted of five trials with at least five jumps per trial. Walking and stair duration depended on completion of the prescribed route.	A researcher accompanied each participant and recorded the start and end of individual activities using a smartphone labeling application.
**HAPT**	Laboratory conditions.	Participants followed a predefined sequence comprising standing, sitting, lying, walking, stair descent, and stair ascent. They were asked to perform the activities relatively freely within the prescribed sequence.	The complete sequence lasted approximately 192 s and contained repeated walking and stair bouts. Individual static activities lasted approximately 15 s and stair bouts approximately 12 s, with short rest periods separating tasks. Each participant performed the protocol twice.	Activity boundaries were established from the experimental protocol and visual annotation. In the first trial the smartphone was fixed on the left side of the waist; during the second it was positioned by the participant.
**IWSDC**	Structured outdoor field protocol followed by real-road driving.	Participants walked at their usual pace along a predefined route containing level walking and repeated stair ascent and descent. Immediately afterward, participants drove their own vehicle along a predefined route containing highway and city driving.	The walking component comprised five level-walking periods, six stair descents, and six stair ascents and lasted approximately 9–13.5 min. Driving lasted approximately 18–30 min depending on traffic conditions.	Participants clapped three times at the beginning and end of activity segments, producing identifiable acceleration peaks that were subsequently used to establish activity boundaries.
**MobiAct**	Institutional data collection with deliberately relaxed smartphone-placement constraints.	Activity type was prescribed, but participants selected the trouser pocket in which the smartphone was carried and its orientation was not fixed. This was intended to better approximate everyday smartphone use.	Standing and walking were recorded for approximately 5 min; jogging and jumping were repeated in 30-s trials; stair ascent and descent were repeated in shorter trials; chair and vehicle-entry/exit transitions were recorded separately.	Activities were recorded using a dedicated smartphone application. Activity type and trial structure were defined by the acquisition protocol.

**MotionSense**	Controlled repeated-trial protocol.	Participants performed walking, jogging, stair ascent, stair descent, sitting, and standing under the same conditions within each trial. Repeated trials of a given activity were obtained in different locations.	Fifteen trials were collected: three each for walking, stair ascent, and stair descent, and two each for jogging, sitting, and standing.	Activity identity was defined by the prescribed trial. The same trial-specific environment and conditions were used across participants.
**PARDUSS**	Predominantly indoor university environment, with cycling performed outside.	Participants performed walking and running in university corridors and stair ascent/descent in a five-floor building. Sitting, standing, jogging, and cycling were also prescribed. Sensor positions and orientations were fixed throughout data collection.	Each activity was performed for approximately 3–4 min. Measurements at the different body positions were obtained simultaneously.	Activity labels followed the prescribed experimental protocol and were recorded using the study data-collection application.
**RealWorld**	Realistic indoor and outdoor environments rather than a conventional laboratory protocol. Walking was performed through a city, jogging in a forest, and stair climbing at an outdoor tower.	Participants were informed which activity to perform, but activity order, speed, and manner of execution were largely left to them. During sitting and standing, participants could engage in behaviors such as using a smartphone, talking, eating, or drinking.	Most activities were performed for approximately 10 min; jumping was shorter, approximately 1.7 min. Participants stood still briefly before and after individual activities.	Activity execution was recorded by video, providing an additional record of the performed movements and activity periods.
**SpeedBreaker**	Naturalistic road travel in the New Delhi National Capital Region using cars, buses, motorcycles, auto-rickshaws, and cycle-rickshaws.	Driving behavior and road conditions were not experimentally standardized. Smartphone placement varied between drives and included locations such as a trouser pocket, dashboard, area near the gearbox, and rear of the vehicle.	The complete source dataset comprised approximately 678 km of road travel involving 22 drivers and several vehicle types. Recordings extended over complete drives rather than short standardized activity trials.	At least two synchronized smartphones were used during each drive. One recorded accelerometer data, while a second was used by the observer to manually mark speed-breaker crossings and their type.
**SisFall**	Controlled university protocol conducted in classrooms and open areas of a university coliseum.	Younger participants performed activities of daily living and simulated falls. Older participants performed only selected activities of daily living; falls and several higher-risk activities were excluded on safety grounds. Falls were performed on safety mats.	Activities had predefined numbers of repetitions and durations. Walking and jogging trials lasted approximately 100 s, stair activities consisted of repeated approximately 25-s trials, and several transitional activities were performed in repeated short trials. Total recording time was approximately 1.5 h per older participant and 3.5 h per younger participant.	Activity identity followed the predefined study protocol. The protocol was supervised and modified for older participants according to physician recommendations.
**SPADES**	Combination of controlled ambulation and a broader indoor/outdoor multi-activity protocol.	The source protocol was designed to expose the accelerometers to both rhythmic and non-rhythmic movements spanning a broad range of frequencies and amplitudes. During the multi-activity component, participants were instructed which activity to perform but were not instructed how to perform it. The ambulation component	Activities in the multi-activity component were generally performed for approximately 2–4 min. The ambulation protocol comprised successive speed conditions.	A researcher recorded the start and stop times of the individual activities.

		included walking and running across progressively increasing speeds.		

1.2 Physical activity estimates vs. sampling frequency

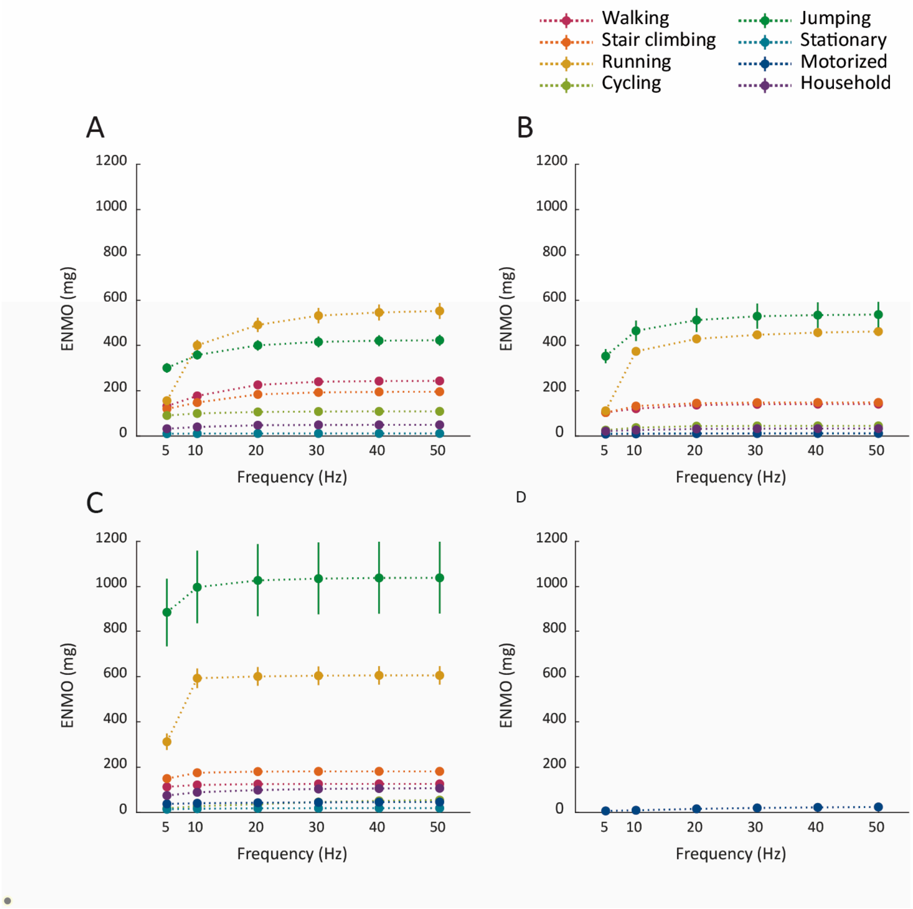

Figure S1. Euclidean Norm Minus One (ENMO) estimates across accelerometer sampling frequencies. ENMO estimates are shown by sensor location: (A) lower limb, (B) trunk, (C) upper limb, and (D) unconstrained placement. Colors distinguish physical activity types. Variation across sampling frequencies indicates the extent to which sampling rate influences estimated movement intensity.

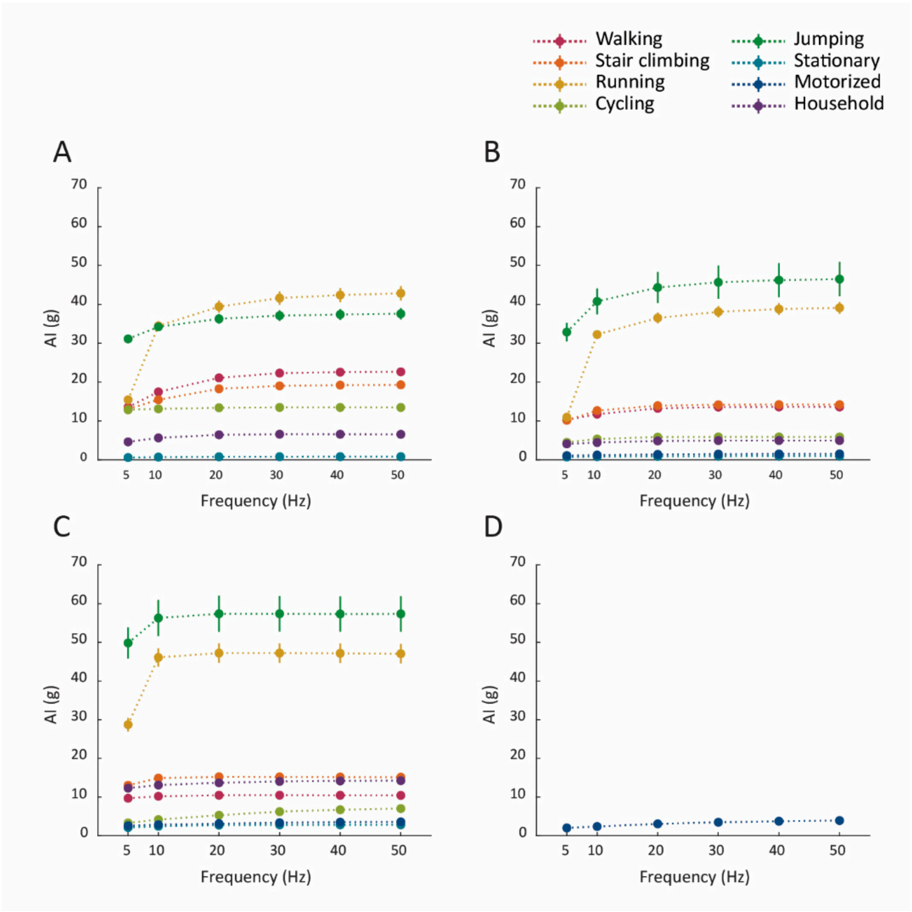

Figure S2. Activity Index (AI) estimates across accelerometer sampling frequencies. AI estimates are shown by sensor location: (A) lower limb, (B) trunk, (C) upper limb, and (D) unconstrained placement. Colors distinguish physical activity types. Variation across sampling frequencies indicates the extent to which sampling rate influences estimated movement intensity.

1.3 Error in estimation of physical activity metrics

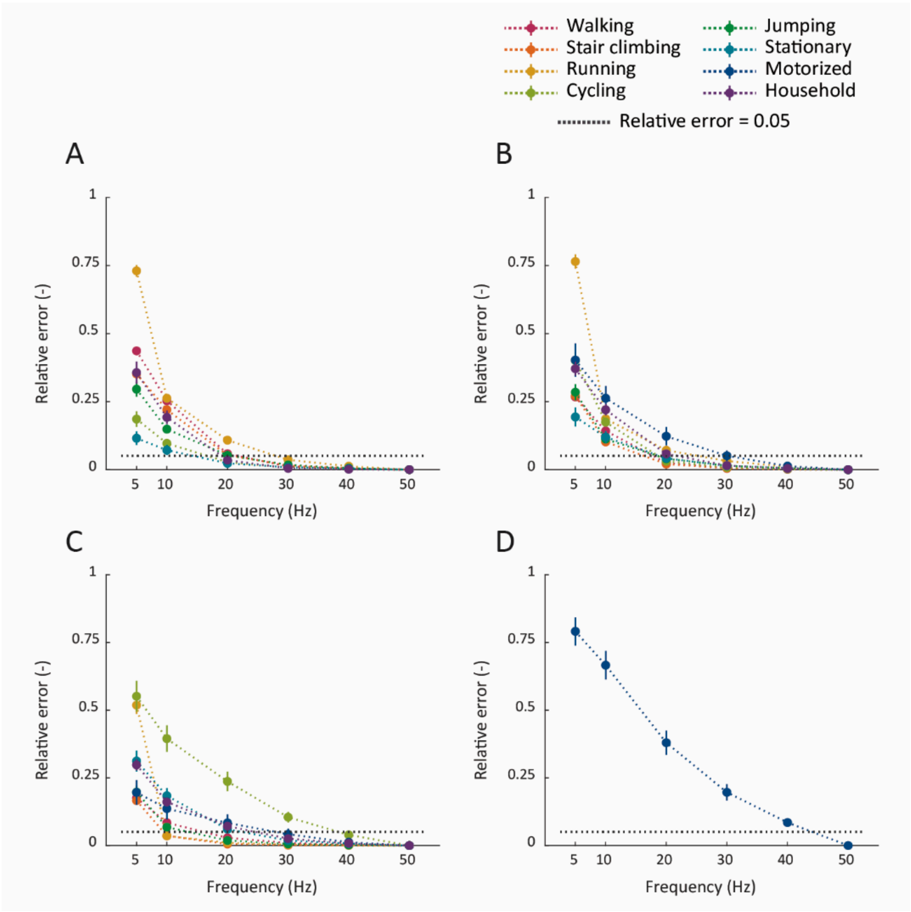

Figure S3. Relative error in ENMO across accelerometer sampling frequencies. Relative error was calculated against the corresponding 50-Hz estimates. Results are stratified by sensor location: (A) lower limb, (B) trunk, (C) upper limb, and (D) unconstrained placement. Dots indicates mean values, while solid vertical lines indicate 95% CI. Colors distinguish physical activity types. Lower values indicate closer agreement with the 50-Hz reference. Dotted black horizontal line indicate 0.05 error criterion.

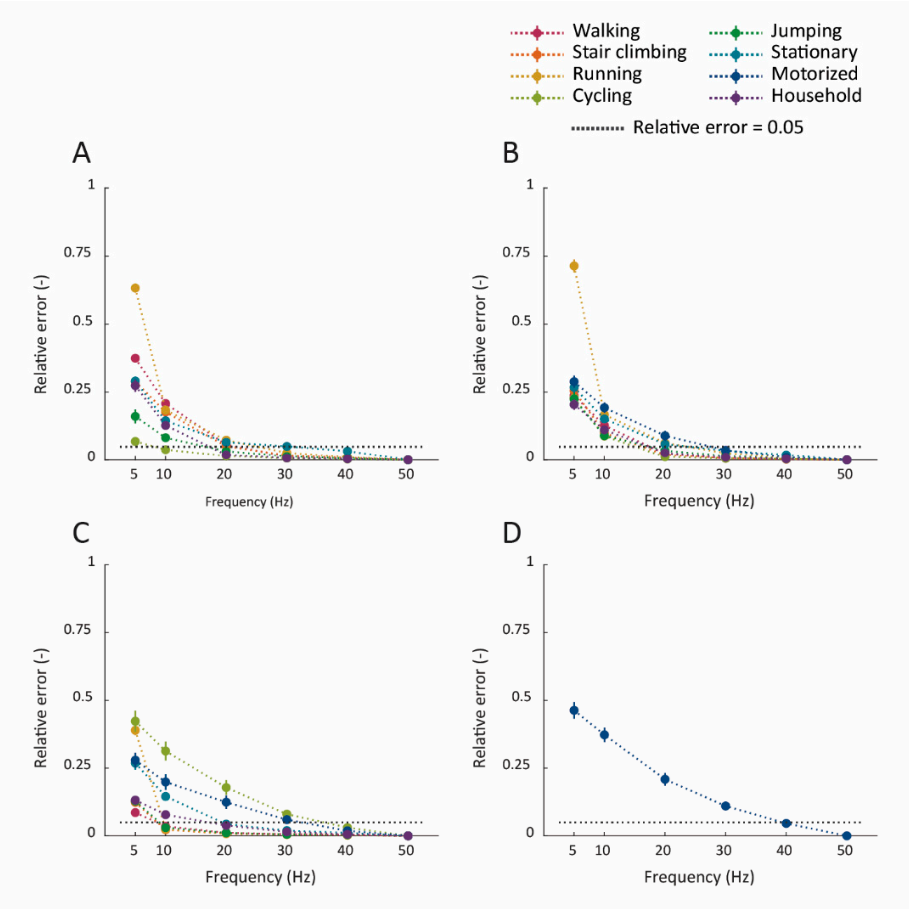

Figure S4. Relative error in AI across accelerometer sampling frequencies. Relative error was calculated against the corresponding 50-Hz estimates. Results are stratified by sensor location: (A) lower limb, (B) trunk, (C) upper limb, and (D) unconstrained placement. Dots indicates mean values, while solid vertical lines indicate 95% CI. Colors distinguish physical activity types. Lower values indicate closer agreement with the 50-Hz reference. Dotted black horizontal line indicate 0.05 error criterion.
